# Prioritizing human-AI collaboration in healthcare: the TRIAD framework for trustworthy governance, real-world, and integrated adaptive deployment

**DOI:** 10.1186/s40779-026-00684-w

**Published:** 2026-02-10

**Authors:** Jia Li, Zi-Chun Zhou, Zhen-Chang Wang, Han Lv

**Affiliations:** 1 Department of Radiology, Beijing Friendship Hospital, Capital Medical University, Beijing, 100050 China; 2 Department of Health Data Science, School of Medical Technology, Capital Medical University, Beijing, 100050 China; 3 Precision and Intelligence Medical Imaging Lab, Beijing Friendship Hospital, Capital Medical University, Beijing, 100050 China

**Keywords:** TRIAD, Artificial intelligence (AI), Human-AI collaboration, Trust mechanisms, Clinical decision support

## Abstract

Artificial intelligence (AI) and big data are reshaping the healthcare landscape. However, clinical value depends on how well systems augment clinicians and fit into routine workflows. To this end, we introduce the TRIAD framework: trustworthy governance, real-world clinical value, and integrated adaptive deployment, to guide the development, validation, and deployment of clinical AI. TRIAD requires explicit data provenance and intended use, fairness auditing, and calibrated uncertainty. This framework evaluates the human-AI team in real workflows using team-level metrics, including accuracy, safety, workload, and patterns of acceptance, editing, and overriding. Deployment proceeds via staged rollouts with preregistered guardrails and continuous monitoring of performance and subgroup impact. TRIAD views intelligence as a property of the human-AI team rather than the AI model alone. Aligning governance, evaluation, and deployment around clinicians and patients enables durable gains in safety, equity, efficiency, and experience, thereby elevating clinical value.

## Background

Artificial intelligence (AI) and big data have reshaped the healthcare landscape, showing promising capabilities in assisting diagnosis, treatment planning, and clinical decision-making. Meanwhile, the growth in medical data has established a foundation for increasingly sophisticated AI applications, such as diagnostic support and personalized treatment recommendations [1]. In this way, medical imaging plays a central role, with AI tools demonstrating remarkable capabilities in areas ranging from radiograph interpretation to complex neuroimaging analysis [2–5].

AI has achieved expert-level performance across a broad range of medical specialties by mastering a range of clinical tasks, fundamentally challenging traditional diagnostic paradigms. In diagnostic classification, systems now match dermatologist-level performance in skin cancer classification and reduce both false positives and negatives in prostate cancer screening [6, 7]. Abnormality detection shows comparable gains, reaching 90.1% area under the curve (AUC) for diabetic retinopathy [8] and near-human performance satisfying the World Health Organization’s suggested accuracy profile for tuberculosis [9]. The scope extends to complex assessment and prediction, with AI generating diagnostic-quality Gleason prostate cancer classification [10] and, impressively, identifying atrial fibrillation from electrocardiograms (ECGs) that appear normal to the human eye [11]. In addition to these specific tasks, AI applications are evolving to handle multimodal clinical reasoning, from mapping brain aging factors [12] to using large language models (LLMs) for clinical decision support [13]. Taken together, these developments indicate a clear shift: AI is rapidly evolving from a specialized tool into a versatile diagnostic powerhouse.

Despite technological achievements, the critical challenge lies not in algorithmic performance but in meaningful clinical integration. As healthcare institutions pursue digital transformation, the pivotal question is more than how well AI can perform, but how it can best collaborate with clinicians to enhance care [14, 15]. The key challenge in implementation has moved from improving model accuracy to enabling effective human-AI teamwork.

To this end, we introduce the TRIAD framework, aligning trustworthy governance (T), real-world clinical value (R), and integrated adaptive deployment (IAD), strive to transform the isolated algorithms into integrated, auditable systems that can earn clinical trust and deliver sustained impact.

## The critical importance of human-AI collaboration: from current evidence to future imperatives

### The landscape of human-AI collaboration: prevailing scenarios, modes, and metrics

**Fig. 1:**
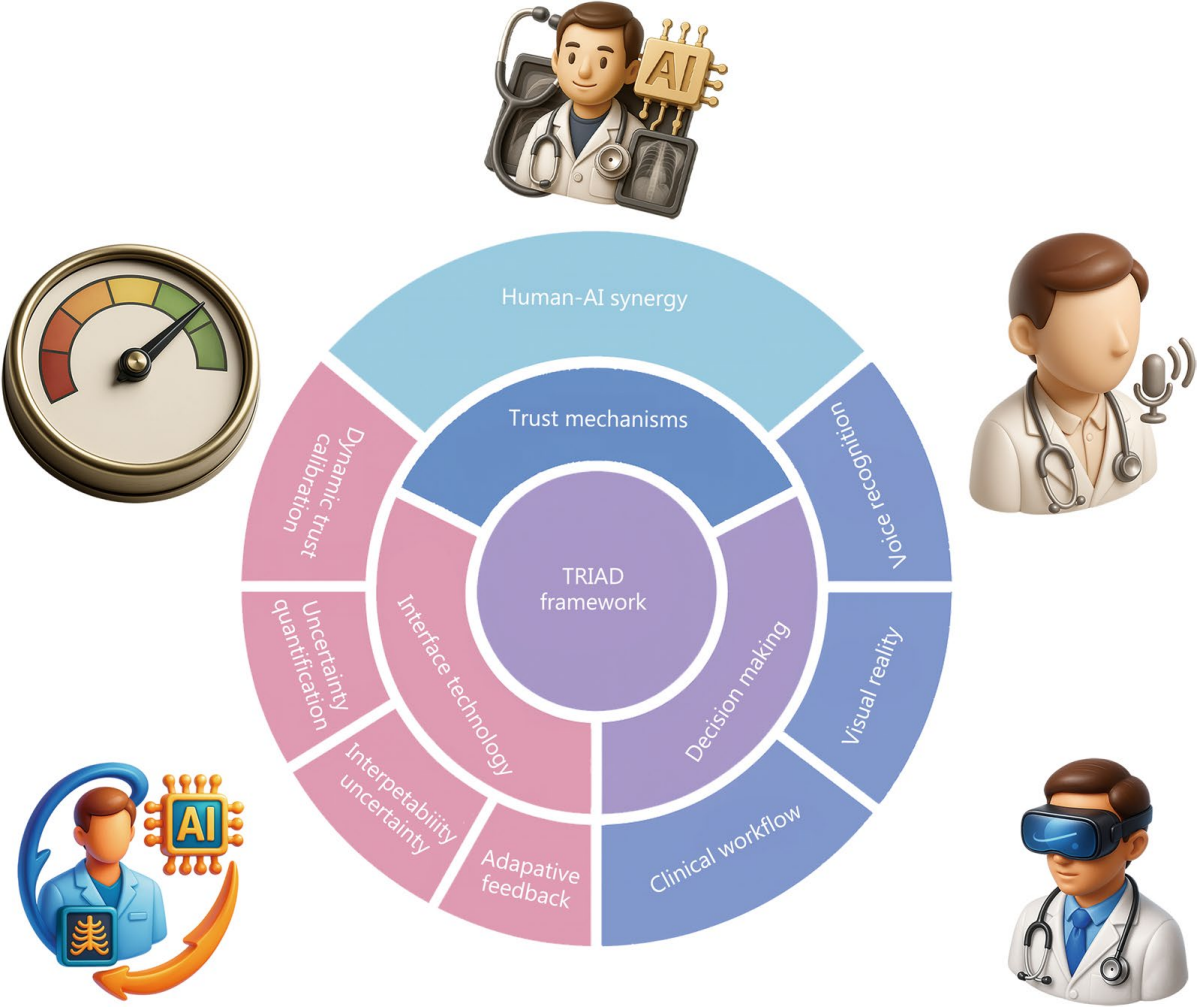
Illustration of a human-AI collaboration system in medicine in the TRIAD framework. TRIAD aligns technical design with trust-building, so AI acts as a complementary teammate, using calibrated uncertainty, adaptive interfaces, and continuous feedback to enhance clinician judgment, safety, and efficiency. AI artificial intelligence, TRIAD trustworthy governance, real-world clinical value, and integrated adaptive deployment

The paradigm of medical AI is shifting toward an augmentative philosophy, as outlined in Fig. 1. A growing body of evidence demonstrates that human-AI collaboration can yield superior clinical performance and efficiency compared to either entity operating alone. This advantage arises from the deep complementarity between the scalable pattern recognition of AI and the contextual, holistic judgment of clinicians. A landscape of representative studies is summarized in Table 1 [16–29].

**Table 1:** A landscape of recent human-AI collaboration studies in healthcare

Scenario/Domain	Study and design	Key outcome	References
Chest radiography reporting	Generative AI drafts, reader study (multireader)	Faster reporting; Improved sensitivity without degrading overall quality	[16]
Chest X-ray report generation	Prospective reader study with AI (Flamingo-CXR)	AI-generated reports are often equivalent or preferred to human reports; Clinician-AI collaboration further improves preference rates	[17]
Chest X-ray impressions	GPT-4 evaluation, blinded reader study	Comparable preference/equivalence in impressions	[18]
Population screening (mammography)	MASAI prospective paired-reader non-inferiority	“1 radiologist + AI” non-inferior to double-reading;	[19]
Chest CT interpretation	Prospective randomized assistance study	Reduced interpretation time	[20]
CXR nodule detection	Randomized controlled trial in screening cohort	Improved detection with AI assistance	[21]
Human-AI collectives (diagnostic vignettes)	Mixed human + LLM ensembles	Outperform either humans or LLMs alone	[22, 23]
Pathology copilot	Multimodal generative assistant for pathologists	Expert preference; Enhanced complex reasoning support	[24]
Digital pathology adoption	Review/world tour	Context for integration and workflow	[25]
Reader performance and efficiency	AI-aided CXR interpretation	Modest gains; Context-dependent effects	[26]
Automation bias (safety)	ECG reading with automated diagnoses	Automation bias reduces accuracy/increases over-trust	[27]
Human-AI interaction in screening	ScreenTrustCAD analysis	Differences in recall/PPV for AI-flagged vs. human-flagged	[28]
Safe auto-reporting	Identifying unremarkable CXRs for automated reporting	Maintained safety with workload savings	[29]

As evidenced by these trends, AI’s role is evolving from purely instrumental to fundamentally complementary. To guide this transition, we now propose a structured framework for building the future of medical AI on deep and durable collaboration. *AI* artificial intelligence, *LLM* large language model, *MASAI* mammography screening with artificial intelligence, *CT* computed tomography, *CXR* chest radiograph, *ECG* electrocardiogram, *CAD* computer-aided detection/diagnosis, *PPV* positive predictive value, *GPT-4* generative pre-trained transformer 4

First, a primary focus of collaboration lies in augmenting high-volume diagnostic workflows to improve efficiency and accuracy. In reporting workflows, for instance, AI-generated drafts have been shown to significantly reduce radiologist reading times and increase sensitivity for specific findings without degrading overall report quality [16]. Furthermore, studies utilizing blinded pairwise comparisons show that AI-generated reports can be preferred or rated as equivalent to human-only reports in certain scenarios, and that clinician-edited AI drafts result in even higher quality outputs [16–18]. In the realm of screening and triage, the landmark MAmmography screening with AI (MASAI) trial demonstrated that a “1 radiologist + AI” not only improved cancer detection but also substantially reduced the radiologists’ workload for initial reads compared to the standard 2-radiologist double-reading [19]. This model, where AI acts as a tireless and consistent second reader, supports safer and more sustainable staffing configurations in resource-strained environments.

Second, beyond the tasks optimizing the current clinical workflow, human-AI collaboration is extending the edge to complex, open-ended diagnosis. Research on “hybrid collectives” shows that ensembles of humans and LLMs can outperform either group alone by leveraging their complementary, weakly correlated error patterns [22, 23]. Evidence for AI as a reliable partner, rather than merely a decision aid, comes from multimodal “copilots”, notably vision-language assistants enabling colleague‑style dialogue for pathologists, which experts strongly prefer for complex tasks [24], alongside broader digital pathology adoption contexts [25]. These advanced systems convey a clear trend toward more dynamic and interactive partnerships.

Third, the evidence also highlights the serious sociotechnical challenges: safety, bias, and human factors in collaborative settings. Studies examining safety have shown that while well-designed AI can offer modest gains, a systematically biased AI can impair clinician accuracy, and that simple explanations may not be sufficient to prevent these adverse effects [30, 31]. More specifically, research in teledermatology has revealed that while AI support can improve overall diagnostic accuracy, it can simultaneously widen performance gaps across subgroups (e.g., for different skin tones), necessitating vigilant fairness monitoring [32]. These findings serve as a critical reminder that trust cannot be assumed and that the risks of automation bias are real and consequential.

The evaluation of these diverse collaborations has also matured, moving beyond simple performance indicators. As shown in Table 1 [16–29], while traditional metrics like area under the receiver operating characteristic curve (AUROC), sensitivity, and reading time remain essential, the field is increasingly adopting more nuanced, human-centric measures. These include blinded preference rates [18], clinically significant error rate analysis [33], task load and burnout scales [31], and error-complementarity matrices [32]. This shift in evaluation offers a broader view that the success of human-AI collaboration is not just about technical accuracy, but about its holistic impact on the clinician, the workflow, and ultimately, the patient.

### The limitations of the current research paradigm

Despite promising results, real-world evidence shows modality- and context‑specific gaps. In dermatology, clinicians significantly outperformed a top-performing AI by integrating patient history and non-visual cues unavailable to the algorithm [34]. Similarly, in a hospital vignette study, exposure to systematically biased AI recommendations reduced physicians’ diagnostic accuracy, even when explanations were provided [35]. This “lab-to-clinic” performance drop is a recurring theme, compounded by a scarcity of robust, prospective, and multi-center randomized controlled trials (RCTs) needed to validate real-world impact and safety at scale. The current evidence base, while encouraging, often reflects an idealized reality rather than the messy, high-stakes environment of actual patient care.

Furthermore, the prevailing research paradigm often dangerously oversimplifies the “human-in-the-loop”, treating clinicians as simple verifiers rather than complex cognitive agents. This overlooks the profound risks of automation bias, where clinicians may uncritically accept incorrect AI suggestions, posing a direct threat to patient safety [27, 36]. Most studies are cross-sectional, which evaluate only a single point in time. As a result, they miss changes in trust and clinician skill decay [36, 37]. Proposed fixes: for example, explainable AI (XAI) is not a cure-all; it can create a false sense of security in a flawed model without actually improving decision-making [37]. These gaps, spanning from flawed evaluation paradigms to the neglect of human cognitive factors, collectively underscore the urgent need for a more holistic framework to guide the development of truly resilient and trustworthy human-AI collaboration.

## The TRIAD framework for trust-collaborative system

**Fig. 2:**
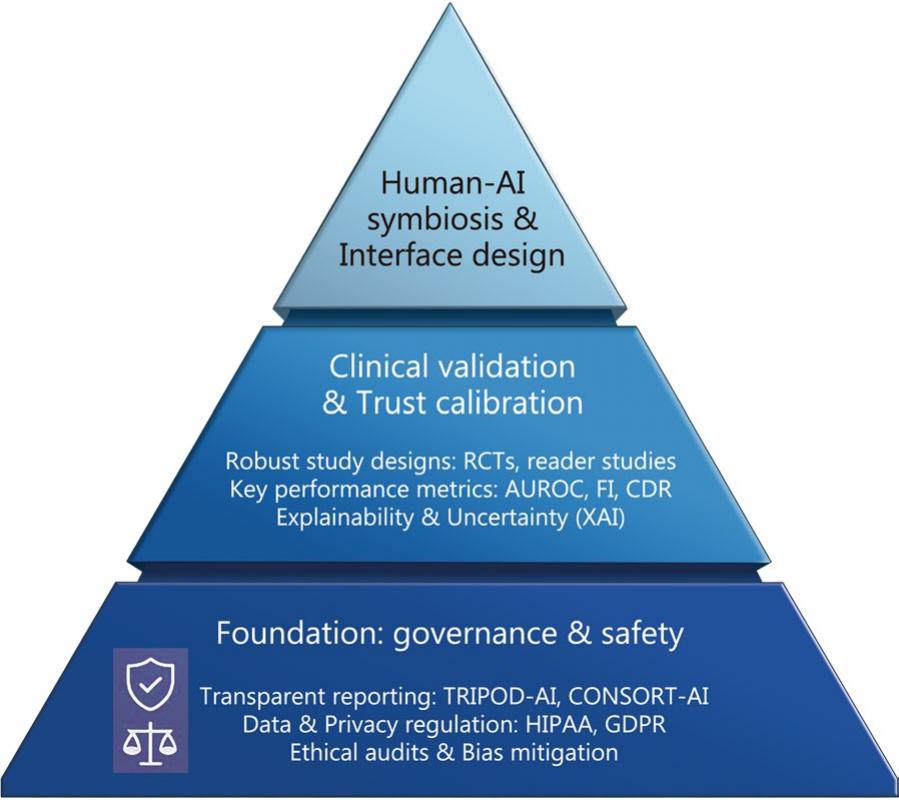
The TRIAD framework shown as a three‑layer pathway linking governance, real‑world evaluation, and adaptive deployment. Transparent data stewardship and ethical oversight form the foundation for workflow‑native human-AI systems that deliver reliable, sustained improvements in patient care. AI artificial intelligence, RCTs randomized controlled trials, AUROC area under the receiver operating characteristic curve, TRIPOD-AI transparent reporting of a multivariable prediction model for individual prognosis or diagnosis-artificial intelligence, CONSORT-AI consolidated standards of reporting trials-artificial intelligence, HIPAA health insurance portability and accountability act, GDPR general data protection regulation

To bridge the gap between AI’s potential and real-world utility, we introduce the TRIAD framework: a 3-layer pyramid for developing, validating, and deploying trustworthy human-AI collaboration (Fig. 2). TRIAD charts a disciplined path from promise to practice. T secures the normative and technical ground on which anything clinical can stand; R tests whether human + AI truly improves care in the intended setting; IAD sustains appropriate trust by merging calibrated confidence with workflow‑native interfaces and safeguards. The 3 layers are intentionally connected: governance guides meaningful validation, and validation, in turn, shapes deployable systems that clinicians can use and trust in routine care. As a summary, possible operational priorities and monitoring indicators are summarized in Table 2 [16, 19, 20, 27–30, 33, 38–51].

**Table 2:** TRIAD framework quick reference for clinical AI deployment

TRIAD	Priority area	Recommended artifacts	References
T: trustworthy governance	Data provenance & Privacy	Source tracking; Data lineage; HIPAA/GDPR compliance; De-identification QA	[39, 40]
	Fairness & Bias management	Subgroup performance deltas; Bias audits; Equity dashboards	[38, 41, 43]
	Transparent reporting	Model card + CONSORT-AI/TRIPOD-AI/SPIRIT-AI adherence	[44–46]
	Risk & Lifecycle safety	Hazard analysis; CAPA; Change control; Post-market surveillance	[42, 47, 48]
R: real-world clinical value	Workflow-native trials	Time to report; Turnaround; Miss-sensitive endpoints	[19, 20]
	Team effectiveness	Human-AI error complementarity; Override/accept/edit rates	[28]
	Safety at scale	Clinically significant error rate; Safe auto-reporting	[29]
	Screening performance	Workload reduction without sensitivity loss	[19]
IAD: integrated adaptive deployment	Calibrated uncertainty	Well-calibrated scores; Abstention/gray-zone routing	[16, 28, 29]
	Human-factors safeguards	Secondary review triggers; Reversible actions; Audit trails	[27, 30]
	Burnout and burden	Task load (NASA-TLX), documentation time, after-hours EHR	[33]
	Interface modalities	Interface modalities	[49–51]

*AI* artificial intelligence, *TRIAD* trustworthy governance, real‑world clinical value, and integrated adaptive deployment, *HIPAA* health insurance portability and accountability act, *GDPR* general data protection regulation, *CONSORT‑AI* consolidated standards of reporting trials-artificial intelligence, *TRIPOD‑AI* transparent reporting of a multivariable prediction model for individual prognosis or diagnosis-artificial intelligence, *SPIRIT‑AI* standard protocol items recommendations for interventional trials-artificial intelligence, *CAPA* corrective and preventive actions, *NASA-TLX* NASA task load index, *EHR* electronic health record, *QA* question and answer

### T: trustworthy governance and ethics

The framework rests on trustworthy governance and ethics, which must precede any clinical deployment of medical AI. T unifies traceable data provenance and privacy, rigorous labeling and subgroup fairness checks, and transparent reporting with life-cycle safety thinking. In health systems, this maps to ethics/institutional review board (IRB) oversight, de-identification and privacy impact work, and conformance to established reporting and safety frameworks. We therefore recommend a pragmatic starting stance: make provenance and privacy explicit, audit fairness where it matters, and publish concise, standards‑aligned documentation, so governance becomes a living contract that clinicians and patients can read, trust, and hold to account.

Firstly, rigorous data governance is essential, ensuring that training and validation data are high-quality, representative, and handled with strict adherence to privacy regulations like health insurance portability and accountability act (HIPAA) in the United States and general data protection regulation (GDPR) in Europe [39, 40]. Secondly, ethical and regulatory compliance involves proactive detection and mitigation of algorithmic bias to ensure fairness across diverse patient populations, a challenge highlighted in numerous studies and frameworks [38, 41, 43]. Finally and crucially, this layer mandates transparent reporting standards. Following guidelines such as consolidated standards of reporting trials (CONSORT)-AI [44], transparent reporting of a multivariable prediction model for individual prognosis or diagnosis (TRIPOD)-AI [45], and standard protocol items: recommendations for interventional trials (SPIRIT)-AI [46] for trials, and aligning with National Institute of Standards and Technology (NIST) AI risk management framework (RMF) [42], ISO 14971 (medical devices, application of risk management to medical devices) [47] and IEC 62304 (medical device software, software life cycle processes) [48] for risk and lifecycle management, these frameworks compel developers to transparently document data sources, model architecture, performance, and limitations, making the entire development process reproducible and accountable. Together, these elements form a foundation that makes an AI system technically sound, ethically responsible, and a safe candidate for clinical evaluation. The goal is shared clarity: where data come from, for whom the model serves well or poorly, and how its limitations and updates are communicated to clinicians and patients.

### R: real‑world clinical value and cognitive synergy

R is the proving ground of the framework: it asks whether a deliberately designed human-AI team, with a clear division of cognitive labor, outperforms either party alone in the workflow where care actually happens. Real-world anchors show this is achievable population mammography, where AI‑supported single reading (1 radiologist + AI) matched the cancer-detection performance of double reading while reducing workload; prospective randomized studies in chest computed tomography (CT) that shortened interpretation time; and chest radiography pathways where AI-drafted reports cut reporting time without degrading overall quality [19, 20]. These gains arise from complementary, weakly correlated errors that hybrid teams can exploit; the point is not higher AUROC in isolation, but measurable improvement in care and capacity at the bedside [29].

We recommend pre-specifying the collaboration agreement: what the AI sees first, what escalates, and what triggers human override-then validating outcomes that clinicians and managers can feel. This may typically include routing thresholds (tH/tL) and a gray zone; a pre-registered statistical analysis plan; and endpoints beyond AUROC: sensitivity where misses matter, time, and turnaround in busy services, clinically significant error profiles, blinded preference when 2 reports read equally well, and inter‑reader consistency and subgroup fairness checks [28]. In short, R shifts the emphasis from “can it classify?” to “does this team work better here?”.

### IAD: integrated adaptive deployment

IAD welds calibrated trust to the interface and day-to-day workflow. For this stage, systems become native to picture archiving and communication system (PACS)/radiology information system (RIS)/electronic medical record (EMR) and clinical notes, communicate task-appropriate uncertainty at decision time, highlight the deltas between machine suggestions and human impressions, and apply simple safeguards where risks are asymmetric (for example, a second look, dual sign-off, an easy way to reverse with an audit trail).

The evidence shows both benefits and risks: naively trusted recommendations can depress clinician accuracy and explanations alone may not rescue users from that trap [27, 30]; yet workflow-native assistants that meet clinicians where they work can lighten administrative and cognitive load-ambient AI scribes, for example, have been associated with lower burnout and less after-hours documentation in multi-site evaluations [33]. A similar “hands-free, eyes-up” trajectory is visible in procedures: augmented-reality guidance has improved the accuracy of pedicle screw placement in a pilot study, strengthening the case for carefully governed overlays in high-stakes settings [49]. Interactivity is therefore the conduit of trust-moving from clicks to conversation and, at the frontier, to neural interfaces. Recent work decoding speech from neural signals at conversational rates suggests future channels for hands‑free, attention‑friendly clinician-AI interaction [50, 51].

## Future directions: toward symbiotic medical intelligence

Building upon the TRIAD framework, the next phase is a shift from tool-centric AI to symbiotic medical intelligence-clinician-AI teams that are reliable, accountable, and measurably better than either alone. The goal is not merely higher stand-alone accuracy, but durable gains in safety, equity, efficiency, and clinician experience. Trust grows when governance is transparent, real-world benefits are shown, and the system is deployed in a way that matches routine clinical practice.

In this view, intelligence is a property of the human-AI system, sustained by transparent provenance, calibrated recommendations, and feedback loops that keep performance legible over time.

Prospectively, progress will depend on co-evolution: clinicians and AI systems learning from each other within governed, data‑driven routines. Evaluation should prioritize team-level outcomes and longitudinal monitoring, while incentives and education cultivate “teaming” skills and shared responsibility. As interaction between physicians and AI naturalized, clinical practice can move toward attentive, humane, and scalable care-raising the ceiling on quality without eroding judgment. Success will be defined by uplift to human expertise and system resilience, not substitution.

## Conclusions

We introduced the TRIAD framework to center clinical AI on human-AI teamwork. TRIAD links 3 pillars across the lifecycle: trustworthy governance, real-world clinical value, and integrated adaptive deployment. By treating intelligence as a property of the human-AI team, TRIAD helps shift from model accuracy to sustained improvements in safety, equity, efficiency, and clinician experience.

## Data Availability

Not applicable.

## Abbreviations

AI: Artificial intelligence
AUC: Area under the curve
AUROC: Area under the receiver operating characteristic curve
ECG: Electrocardiogram
EMR: Electronic medical record
GDPR: General data protection regulation
HIPAA: Health insurance portability and accountability act
LLM: Large language model
MASAI: MAmmography screening with artificial intelligence
NIST: National Institute of Standards and Technology
RMF: Risk management framework
PACS: Picture archiving and communication system
PPV: Positive predictive value
RCT: Randomized controlled trial
RIS: Radiology information system
SPIRIT-AI: Standard protocol items: recommendations for interventional trials-artificial intelligence
TRIPOD-AI: Transparent reporting of a multivariable prediction model for individual prognosis or diagnosis-artificial intelligence
TRIAD: Trustworthy governance, real‑world clinical value, and integrated adaptive deployment
